# Systematic review of clinical decision support interventions with potential for inpatient cost reduction

**DOI:** 10.1186/1472-6947-13-135

**Published:** 2013-12-17

**Authors:** Christopher L Fillmore, Bruce E Bray, Kensaku Kawamoto

**Affiliations:** 1 Department of Biomedical Informatics, University of Utah School of Medicine, Salt Lake City, Utah 84112, USA

**Keywords:** Clinical decision support, Clinical costs, Cost effectiveness, Hospital care, Emergency medical care, Health information technology

## Abstract

**Background:**

Healthcare costs are increasing rapidly and at an unsustainable rate in many countries, and inpatient hospitalizations are a significant driver of these costs. Clinical decision support (CDS) represents a promising approach to not only improve care but to reduce costs in the inpatient setting. The purpose of this study was to systematically review trials of CDS interventions with the potential to reduce inpatient costs, so as to identify promising interventions for more widespread implementation and to inform future research in this area.

**Methods:**

To identify relevant studies, MEDLINE was searched up to July 2013. CDS intervention studies with the potential to reduce inpatient healthcare costs were identified through titles and abstracts, and full text articles were reviewed to make a final determination on inclusion. Relevant characteristics of the studies were extracted and summarized.

**Results:**

Following a screening of 7,663 articles, 78 manuscripts were included. 78.2% of studies were controlled before-after studies, and 15.4% were randomized controlled trials. 53.8% of the studies were focused on pharmacotherapy. The majority of manuscripts were published during or after 2008. 70.5% of the studies resulted in statistically and clinically significant improvements in an explicit financial measure or a proxy financial measure. Only 12.8% of the studies directly measured the financial impact of an intervention, whereas the financial impact was inferred in the remainder of studies. Data on cost effectiveness was available for only one study.

**Conclusions:**

Significantly more research is required on the impact of clinical decision support on inpatient costs. In particular, there is a remarkable gap in the availability of cost effectiveness studies required by policy makers and decision makers in healthcare systems.

## Background

Healthcare costs are increasing rapidly and at an unsustainable rate in many countries. In the United States, inpatient care is the single largest contributor to national health expenditures, accounting for 31.5% of $2.7 trillion dollars of health expenditures in 2011 [1]. As such, inpatient care is a significant driver of increased health spending. In 2011, the annual spending on hospital care in the U.S. grew 4.3% as compared to 3.9% growth in overall health expenditures [1]. Contributing to the importance of addressing inpatient costs is the fact that reducing these costs has the potential to financially benefit inpatient healthcare organizations regardless of reimbursement models. Traditional episode-of-care payment systems (for example, Medicare's inpatient prospective payment system), bundled payments systems, and comprehensive payment systems (embodied in accountable care organizations) are all examples of reimbursement models under which healthcare organizations stand to benefit from reducing inpatient costs [2].

Clinical decision support (CDS) represents a promising approach to both improving outcomes and decreasing costs [3]. Several past systematic reviews have examined outcomes related to clinical decision support systems in the inpatient setting, but few have focused on the impact of CDS on inpatient costs specifically [4-9]. One review published in 2006 evaluated cost as an outcome [10]. However, that review was focused on health information technology (IT) in general rather than CDS specifically. Moreover, it only included studies published through January 2004. A second, more recent review on CDS included cost outcomes but was limited to studies with a randomized trial design [11]. The design and timing of these two reviews potentially excluded relevant CDS intervention trials. In particular, non-randomized research designs are commonly used to evaluate CDS interventions.

Given the importance of limiting the growth of inpatient costs and the potential benefit of CDS, we sought to (i) inclusively identify promising interventions that could serve as models for more widespread implementation and to (ii) identify gaps in the literature warranting further research. As such, we systematically reviewed both randomized and non-randomized trials of CDS systems with the potential to reduce inpatient or emergency department (ED) costs.

## Methods

### Data source

Using a search strategy adapted from a previous systematic review [7], we searched MEDLINE through July 18, 2013. The latest search was performed on that date. We used a combination of the following search terms: *decision support systems, clinical; decision-making, computer-assisted; computerized decision support; reminder systems; guideline adherence;* and *medical informatics.* Details of the search strategy are available in Appendix I. Search results were limited to human subjects and the English language.

### Inclusion and exclusion criteria

We defined a CDS system as a system designed to directly aid in clinical decision making, in which characteristics of individual patients are matched to a knowledge base for the purpose of presenting patient-specific assessments or recommendations to clinicians [4]. Inclusion criteria were as follows: peer-reviewed primary manuscript; clinical trial of a CDS system in an inpatient or emergency department (ED) setting; and use of either cost or a proxy measure for cost (e.g., length of stay; see Data Extraction section for full list) as an outcome metric. Of note, we opted to include studies in an ED setting, as these costs often become part of inpatient costs because many ED patients are admitted to the hospital. Exclusion criteria were as follows: non-English manuscript; or use of CDS in the control group.

### Study selection

Titles and abstracts from retrieved references were evaluated by a single reviewer to determine potential inclusion eligibility. The full texts of studies that appeared to be potentially eligible were then evaluated by the same reviewer. Final inclusion determinations were made using the full texts. In cases where a study’s inclusion status was unclear upon review by the primary reviewer, the authors jointly reviewed the study and made a consensus decision.

### Data extraction

Data extraction was performed by a single reviewer using a standardized form. Any issues involving uncertainty were resolved through author consensus. For each article that met inclusion criteria, data were extracted on setting, trial design, intervention, and trial results. Setting was assigned as one of three categories: ED, ICU, or hospital. A hospital setting typically included inpatient wards, but could also include a combination of wards, ED, ICU, or surgical settings. Trial design was assigned based on categories defined by the Cochrane Effective Practice and Organization of Care Group [12]. Design categories included randomized controlled trials (RCTs), non-randomized controlled trials (NRCTs), controlled before-after studies (CBA), and interrupted-time-series studies (ITSs).

Abstracted trial results included outcomes with potential cost saving implications, whether costs were directly measured, whether there was a statistically and clinically significant improvement in cost or in a proxy measure, and whether the study could be considered a cost effectiveness study. Specifically, measures with potential cost saving implications consisted of direct cost measures or other proxy measures with cost ramifications. Proxy cost measures included length of stay, readmission rates, resource utilization metrics (e.g., imaging studies), adverse events, and process measures correlated with adverse events. A change in a cost or proxy measure was decided a priori to be the primary outcome measure. Clinical significance of results was determined by author consensus. To be considered a cost effectiveness study, the study must have accounted at least for the personnel costs included in developing and deploying the intervention. For commercial CDS systems, at least the cost of licensing the software must also have been considered.

### Data analysis

Extracted data were analyzed and presented in table form and narrative summary. Additionally, significant themes, trends, and patterns were noted and discussed. To better understand the potential relationship between study outcomes and potential explanatory factors, Fisher’s exact test of independence was conducted with the independent variable being a statistically and clinically significant improvement in an actual or proxy cost measure. One of the potential explanatory variables examined was study quality, with CBA trials and NRCTs conducted at single sites distinguished from studies that utilized more rigorous study designs. Other potential explanatory variables examined included study setting and clinical domain. For the purposes of this analysis, clinical domains with two or fewer studies were combined into a single category. A p-value of < 0.05 was considered statistically significant.

## Results and discussion

**Figure 1:**
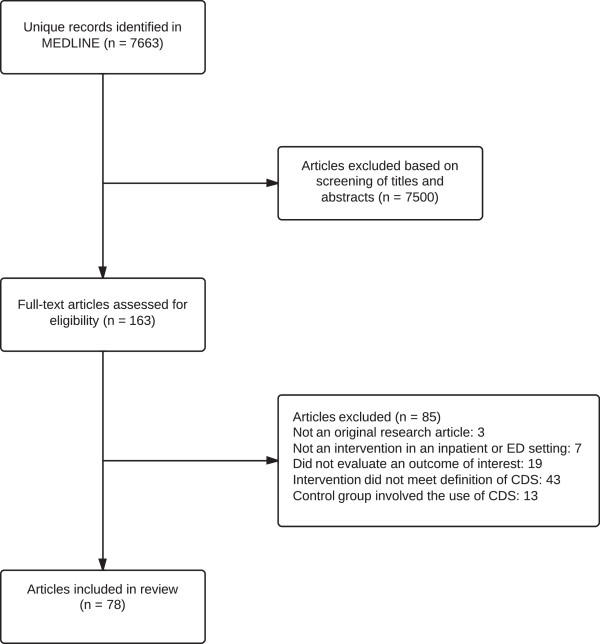
Summary of literature search and selection.

The literature search returned a total of 7,663 unique references. 7,500 references were excluded after screening of titles and abstracts. We reviewed 163 full-text articles, of which 78 [13-90] met criteria for inclusion in the review (see Figure 1). Characteristics of these studies are summarized in Additional file 1: Table S1.

### Study timing

A majority (52.6%) of studies were published during or after 2008 [14,15,18-20,25,26,29,32-34,38-40],[43-47,49,50,55-61,65-67,70,74-76,78],[81,82,85,86,90]. The earliest included study was published in 1989 [54]. This high concentration of studies published between 2008 and 2013 represents large recent growth in the evaluation of inpatient CDS systems and is likely consistent with increasing adoption of health IT generally.

### Study settings

A majority of the studies (55.1%) took place in a general hospital setting ([14-17,19,24-26,28,30,32,33,37],[41-43,46,48-50,52-56,60-62,64,66,67],[69-72,74,76-78,80,85,87,90]. 29.5% of the studies occurred in an ICU setting [18,23,31,34-36,39,40,44,47],[51,57-59,63,65,68,73,79,82],[86,88,89], and 15.4% took place in an ED setting [13,20-22,27,29,38,45,75,81],[83,84].

### Study designs

A large proportion (84.6%) [13-18,20-27,29,30,32-37,39-52,54-58,60],[61,64-70,72-78,82-90] of the studies were quasi-experimental trials, which can be defined as studies that aim to evaluate interventions without the use of randomization [91]. Overall, the most common design used in the studies was the controlled before-after design, wherein researchers used historical controls prior to the implementation of an intervention. Of the 78 studies, 61 (78.2%) were some form of controlled before-after study [13-18,20-27,29,30,32-37,39-51,54,55],[57,60,61,64,65,68-70,72-78,82-90]. Only 12 (15.4%) of the studies were randomized controlled trials (RCTs) [19,28,31,38,53,59,62,63],[71,79-81]. The remaining five studies were interrupted-time-series studies [56,58] and non-randomized controlled trials [52,66,67].

The frequent use of quasi-experimental designs in medical informatics evaluations has been noted previously [91,92], and the results of this review are consistent. Given the overwhelming prevalence of quasi-experimental designs, reviews of CDS systems that only include RCTs are bound to exclude a large portion of the published literature. With respect to this systematic review, a deliberate decision was made to include quasi-experimental studies, as one of the primary goals of this study was to inclusively identify CDS interventions that have the potential for reducing inpatient costs. At the same time, the inclusion of quasi-experimental study designs certainly resulted in the inclusion of studies that are more subject to bias than RCTs.

### Clinical focus

The most common clinical focus targeted by CDS systems in the review was pharmacotherapy, with 53.8% of studies focused on this area [16-18,20,23,24,26,35,37,39],[40,42,43,45,47,48,50,51],[54,57-62,64,65,67-69,71-74,76-79,85],[88-90]. Of the studies focused on pharmacotherapy, the most common areas of specific focus were nephrotoxic drugs (23.8%) [24,41,60,69,76-78,85,90], antibiotics (21.4%) [20,23,26,35,54,62,68,71],[88], and insulin management (14.3%) [39,40,57,59,65,79].

The second most common area of clinical focus was venous thromboembolism prophylaxis, which accounted for 9% of studies in the review [28,30,43,46,53,55,87]. Examples of other clinical areas addressed included blood transfusion management [14,36,80], ventilation management [31,34,63], and radiology utilization [22,29,75].

The significant focus on pharmacotherapy within the included studies may reflect the importance of drug selection within computerized provider order entry (CPOE) systems, which are foundational to CDS in many inpatient settings. 50% [14,16,17,19,22-26,28-30,35-37,41-43],[45,46,48,50,56,58,60,61],[64,66-68,72,73,75,76,80,85],[87,89,90] of the studies overall involved CDS in the context of CPOE, of which 61.5% [16,17,23,24,26,35,37,41],[42,45,48,50,58,60,61,64],[67,68,72,73,76,85,89,90] were focused on pharmacotherapy.

### Cost effectiveness

Only one of the 78 studies (1.3%) was considered to be a cost effectiveness study [33]. This study evaluated the use of a well-known diagnostic decision support system, DXplain, with residents in a teaching hospital. The authors reported that access to DXplain had been provided at no charge for the purposes of the study, but that an annual license would have cost their organization $4,000-$6,000 per year [33]. It is telling that the only study to address cost effectiveness in this review concerned a simple license to a stand-alone, diagnostic CDS system. The majority of the studies in this review dealt with more comprehensive, integrated systems either purchased through vendors or developed locally. Under those circumstances, providing information about cost of development, implementation, or licensing fees is presumably more difficult. However, the near complete lack of this type of information is concerning given the need for such cost-effectiveness information by public policy developers and decision makers within healthcare organizations.

### Direct measurement of cost

10 (12.8%) studies in the review directly measured costs [20,24,33,35-37,62,64,68,71]. Eight of these studies focused on pharmacotherapy [20,24,35,37,62,64,68,71], while the other two addressed management of blood transfusion [36] and general medical diagnosis [33]. These studies included two RCTs [62,71], with the remainder of studies having a quasi-experimental design. Of these studies, five reported a statistically and clinically significant improvement in a cost measure [33,35,36,62,71]. Except in one case as outlined, the cost involved in implementing these interventions was not studied. Because investments in CDS are like any other business investment, having only one side of the financial equation (cost impact) is insufficient for making public policy and business decisions.

### Use of proxy cost measures

87.1% of the studies in the review solely reported proxy measures as indicators of impact on cost [13-19,21-23,25-32,34,38-61,63,65-67,69],[70,72-90]. The most commonly used type of proxy measure in this group was process measures that were associated with adverse events. Of the studies that solely used proxy measures, 52.9% reported this type of measure [15-17,22,25,26,28,30,32,34],[41-43,46,50-54,60,61,66,67,70],[73,74,76-78,80,85-87,89,90]. Examples of other proxy measures reported by these studies included rates of adverse events (reported by 39.7% of studies [16-19,23,25,26,39,40,43,45-47],[53-55,57-59,63,65,67,69,72,73],[79,90]), length of stay (reported by 22.1% of studies [15,19,31,38,44,45,47,49],[57,63,66,67,72,81,82]), resource utilization metrics (reported by 16.2% of studies [13,14,29,38,48,56,75,81],[82,86,88]) and patient charges (reported by 5.9% of studies [21,27,83,84]).

As noted, a strikingly small percentage of the studies directly measured an intervention’s impact on cost. Therefore, in the majority of cases we were left to infer a possible cost savings from non-financial proxy measures. Doing so has some inherent limitations. For example, five studies reported patient charges as an outcome [21,27,33,83,84]. This is not a direct measure of cost, and it can be unclear as to how charges actually relate to cost [93]. We assumed that an institution’s costs were at least proportional to what it charged a patient. However, given that we did not know the actual relationship between costs and charges at any given institution, this assumption suffered from an element of uncertainty.

A second limitation of using proxy measures is illustrated by the conflict of explicit cost measures and proxy measures within the same study. For example, two studies reported no differences in actual measured costs but reported decreases in length of stay [24,64]. For this review, we considered length of stay a reasonable proxy measure for cost. But in these two instances, shorter lengths of stay did not coincide with actual decreased costs. The reverse of this situation was present in two studies, where directly measured costs decreased, but no difference in length of stay was detected [33,62]. It is notable that of the ten studies in the review that directly measured costs, four demonstrated discrepancies between explicit cost measures and available proxy cost measures.

A third limitation of using proxy measures is related to the inconsistent relationship between process measures correlated with adverse events and the actual rates of those adverse events. We considered measures of adverse events an appropriate proxy measure for cost given the potential for these events to necessitate the utilization of additional resources. We went a step further and included process measures correlated with adverse events as proxy measures as well. For example, one study in the review reported the rate of compliance with venous thromboembolism prophylaxis guidelines (a process measure correlated with an adverse event) [30]. Alternatively, another study reported the actual incidence of venous thromboembolism (a measure of an adverse event) [53]. For this review, we made the assumption that an improvement in a process measure associated with an adverse event would be associated with an improvement in the incidence of that adverse event. Decreased incidence of an adverse event, in turn, would be associated with cost savings. However, in one study, process measures correlated with an adverse event were significantly improved, but there was no improvement in the incidence of the actual adverse event [26]. More perplexingly, another study reported improvements in a process measure, no improvement in the correlated adverse event, and a significant improvement in length of stay [67].

### Impact on cost/proxy measures

55 (70.5%) of the studies reported a statistically and clinically significant improvement in a cost or proxy measure [13-18,21-23,25,26,28,30,32,33],[35,36,38-43,45-50,53-57,59-62,65-67,69],[71,73-75,77,78,80,82,84,86-88],[90]. However, when considered in the context of the lack of direct cost measurements, the limitations of proxy cost measures, and the prevalence of quasi-experimental designs, it is difficult to know what level of confidence to place in that finding. On the face of it, CDS does appear to be a promising intervention for reducing inpatient costs. However, further research is clearly needed in order to more concretely characterize the benefits that have been achieved and that might be achieved in the future.

### Relationship between primary study outcome and potential explanatory factors

Table 1 provides the results of Fisher’s exact tests of independence between the primary study outcome and potential explanatory factors. There was a trend towards single-site CBA and NRCT studies having more positive outcomes than more rigorous studies (75% vs. 56%, p = 0.14), which was an expected finding given the bias known in the literature for poorer-quality studies more frequently showing positive outcomes. Study setting did not appear to be significantly associated with study outcomes, whereas clinical domain did appear to have a significant relationship with study outcomes (p = 0.04). In particular, all of the seven studies on VTE prophylaxis and all of the three studies on transfusion had successful outcomes. However, we caution against over-interpreting the implications of these statistical analyses, as the interventions were heterogeneous in nature, and as the proxy outcome measures utilized in these analyses had several important limitations as discussed earlier. The primary finding from our study remains that additional research is required on the true cost implications of CDS in the inpatient setting.

**Table 1:** Study outcomes and potential explanatory variables

***Study outcomes***				
***Study characteristics***	***Overall (N = 78)***	***Non-successful (N = 23)***	***Successful (N = 55)***	***p-value***
**Single site CBA or NRCT**				0.14
**No**	18	8 (44%)	10 (56%)	
**Yes**	60	15 (25%)	45 (75%)	
**Setting**				0.17
**ED**	12	5 (42%)	7 (58%)	
**Hospital**	43	9 (21%)	34 (79%)	
**ICU**	23	9 (39%)	14 (61%)	
**Domain**				0.04
**Pharmacotherapy**	42	12 (29%)	30 (71%)	
**Radiology**	3	1 (33%)	2 (67%)	
**Transfusion**	3	0	3 (100%)	
**VTE Prophylaxsis**	7	0	7 (100%)	
**Ventilation management**	3	3 (100%)	0	
**Other**	20	7 (35%)	13 (65%)	

### Models for more widespread implementation

One of the aims of this study was to identify interventions that were promising for more widespread implementation. As noted, a large proportion of the CDS interventions found in this review are related to pharmacotherapy within the context of CPOE. As organizations continue to adopt or expand CPOE systems with integrated CDS, a potentially promising area to focus on is the management of antibiotics, as three of the five studies that directly measured costs and demonstrated improvement in cost metrics [35,62,71] were focused specifically on the management of antibiotics. Another promising area for implementation is venous thromboembolism prophylaxis, as all seven of the studies with this focus demonstrated improvements in the outcomes of interest [28,30,43,46,53,55,87].

### Limitations

Our study is potentially limited by the use of only one database, MEDLINE, for our search. As a result, there was a risk to exclude relevant articles. However, in a previous systematic review of CDS interventions [7], which searched MEDLINE, CINAHL, and the Cochrane Central Register of Controlled Trials, all 88 of the studies included in the final study sample were indexed and available in MEDLINE. Therefore, we believe that this risk is limited. A further potential limitation of our study is the use of a single reviewer to perform study selection and data extraction. However, any issues involving uncertainty were resolved through author consensus to manage this risk.

## Conclusions

Health IT, and CDS in particular, has been touted for many years as a highly promising strategy for improving clinical care and “bending the cost curve” [94,95]. However, more recent analyses have found that health IT systems such as EHR systems are not having the anticipated benefits in cost reduction [96,97]. This study adds to these concerns that the potential benefits of health IT and CDS are not well grounded in empirical evidence, with only ten studies directly measuring costs and only one actually measuring cost-effectiveness of CDS for inpatient cost reduction.

As healthcare organizations continue to rapidly adopt health IT, leadership within those organizations must decide how to best use limited resources. Presumably, the potential cost savings associated with intervention candidates is a major factor in making those decisions. However, as a discipline, informatics does not appear to be meeting the needs of these healthcare decision makers with regard to CDS, as we have not been providing sufficient, rigorous data related to the cost benefits of CDS interventions in the inpatient setting. Further research with specific attention to cost implications of CDS systems in the inpatient setting is clearly needed.

## Appendix I

### Search strategy details

1. exp Decision Support Systems, Clinical/

2. Decision Making, Computer-Assisted/

3. "computerized decision support".mp.

4. exp Reminder Systems/

5. exp Guideline Adherence/

6. exp Medical Informatics/

7. 5 and 6

8. 1 or 2 or 3 or 4 or 7

9. limit 8 to (english language and humans)

## Competing interests

KK has or is currently serving as a consultant on CDS to the following organizations: the Office of the National Coordinator for Health IT, Partners HealthCare, RAND Corporation, ARUP Laboratories, Inflexxion, Inc., Intelligent Automation, Inc., McKesson InterQual, and ESAC, Inc. KK receives royalties for a Duke University-owned CDS technology for infectious disease management known as CustomID that he helped develop. KK was formerly a consultant for Religent, Inc. and a co-owner and consultant for Clinica Software, Inc., both of which provide commercial CDS services, including through use of a CDS technology known as SEBASTIAN that KK developed. KK no longer has a financial relationship with either Religent or Clinica Software. The other authors have no competing interests to declare.

## Authors’ contributions

All authors contributed to the design of the study. CLF served as the primary literature reviewer. All authors contributed to the data analysis and manuscript preparation. All authors read and approved the final manuscript.

## Authors’ information

CLF was an Air Force flight surgeon and is currently a post-doctoral fellow in biomedical informatics. All authors are physicians, and BEB and KK are faculty members in biomedical informatics.

## Pre-publication history

The pre-publication history for this paper can be accessed here:

http://www.biomedcentral.com/1472-6947/13/135/prepub

## Supplementary Material

Additional file 1: Table S1 Summary of study characteristics. Click here for file
