# Supplementary material for: Systematic review of clinical decision support interventions with potential for inpatient cost reduction
Source: BMC Med Inform Decis Mak. 2013 Dec 17;13:135. doi: 10.1186/1472-6947-13-135 (PMC3878492; doi:10.1186/1472-6947-13-135)
Supplement: Additional file 1: Table S1 — Summary of study characteristics. [file 1472-6947-13-135-S1.doc]

Table S1. Summary of study characteristics

| ***Citation*** | ***Setting*** | ***Scope*** | ***Trial design*** | ***Intervention (control = usual care unless otherwise specified)*** | ***Measures with potential cost saving implications*** | ***Results (intervention vs. control)*** | ***Direct measurement of cost*** | ***Statistically & clinically significant improvement in cost (if measured) or proxy metric*** | ***Cost effectiveness study*** |
| --- | --- | --- | --- | --- | --- | --- | --- | --- | --- |
| Aase O (1999) | ED | 1 site; 493 patients | CBA | CDS using Bayes theorem to assist in diagnosis and triage of patients with acute chest pain | Percent of patients unnecessarily admitted to coronary care unit | 19% vs. 35% (P < 0.05)^#^ | No | Yes | No |
| Adams ES (2011) | Hospital | 1 site; 6785 discharges | CBA | CPOE with CDS intended to support the management of children potentially needing RBC transfusions | Rate of RBC transfusions per patient  Estimated direct cost savings during intervention phase | 0.05 vs. 0.076 (P < 0.03)  $165000 (P = N/A) | No | Yes | No |
| Austrian JS (2011) | Hospital | 1 site; 2087 patients | CBA | CDS system to assist in the early detection of HIT | Proportion of patients tested for HIT antibody within 24 hours of a qualifying drop in platelets  Proportion of patients for whom heparin was discontinued within 24 hours of a qualifying drop in platelets  Proportion of patients above the median length of stay adjusted for comorbidities | 41.9% vs. 31.5% (P = 0.03)  26.5% vs. 21.2% (P = 0.01)  50.3 vs. 49.7 (NS) | No | Yes | No |
| Bates DW (1998) | Hospital | 1 site; 6711 admissions | CBA | CPOE with CDS | Non-intercepted serious medication error rate  Preventable ADE rate  Non-intercepted potential ADE rate | 4.86 vs. 10.7 events/1000 patient days (P = 0.01)  3.88 vs. 4.69 events/1000 patient days (NS)  0.98 vs. 5.99 events/1000 patient days (P = 0.002) | No | Yes | No |
| Bates DW (1999) | Hospital | 1 site;  1817 admissions | CBA | CPOE + CDS with serial improvements of CDS across study periods | Non-missed-dose medication error rate  Non-intercepted serious medication error rate | 26.6 (final period) vs. 142 events/1000 patient days (P < 0.0001)  1.1 (final period) vs. 7.6 events/1000 patient days (P < 0.0003) | No | Yes | No |
| Bertsche T (2010) | ICU | 1 site; 265 patients | CBA | CDS system designed to decrease the incidence of ADEs related to drug-drug interactions in patients prescribed >8 drugs in the ICU setting | Relative risk of patient experiencing at least one ADE due to drug-drug interactions | 25% vs. 44% (P < 0.01) | No | Yes | No |
| Boustani MA (2012) | Hospital | 1 site; 998 patients | RCT | CPOE with CDS to improve the quality of care for adults with cognitive impairment | Length of stay  30-day readmission rate  Hospital-acquired complication rate | 7.7 vs. 6.8 days (NS)  18.6% vs. 16.4% (NS)  47.2% vs. 44.9% (NS) | No | No | No |
| Buising KL (2008) | ED | 1 site; 525 patients | CBA | CDS designed to support adherence to community acquired pneumonia recommendations | Percent of antibiotic prescriptions concordant with recommendations  Average cost of antibiotics per patient | 89.7% vs. 61.9% (P <0.01)  $84.04 vs. $72.07 (P = N/A) | Yes | No | No |
| Buller-Close K (2003) | ED | 1 site; 280 patients | CBA | Guideline-driven electronic charting system that provided recommendations for evaluation and treatment of occupational exposure to blood or body fluids | Median charges per patient | $392 (intervention)vs. $473 (baseline) (95% CI $51 to $161 difference in charges) | No | Yes | No |
| Carton M (2002) | ED | 2 sites; 1836 patients | CBA | CDS system to provide advice on the appropriate use of imaging studies | Proportion of orders that did not conform to guidelines | 26.9% vs. 33.2% (P = 0.0001) | No | Yes | No |
| Chan AL (2006) | ICU | 1 site | CBA | CDS consisting of an automated gentamicin dosing calculator integrated into an ICU CPOE system | Frequency of undesirable serum gentamicin levels | 13.5% vs. 32.7% (P < 0.05)* | No | Yes | No |
| Chertow GM (2001) | Hospital | 1 site; 17828 patients | CBA | CPOE with CDS that provided adjusted default dose and frequency for patients with renal insufficiency | Mean length of stay  Mean total costs  Mean Pharmacy costs | 4.3 vs. 4.5 days (P = 0.009)  $4881 vs. $4968 (NS)  $168 vs. $166 (NS) | Yes | No | No |
| Cho A (2012) | Hospital | 1 site; 463 patients | CBA | CDS system intended to alert physicians to patients at high risk for contrast-induced AKI when ordering contrast-enhanced CT imaging | Percent of at-risk patients who received prophylaxis for contrast-induced AKI  Incidence of contrast-induced AKI among at-risk patients | 55% vs. 25% (P < 0.001)  3% vs. 10% (P < 0.02) | No | Yes | No |
| Cox ZL (2011) | Hospital | 1 site; 216 patients | CBA | CPOE with CDS to assist in the prescription of tobramycin and amikacin | Proportion of initial doses within 10% of the calculated recommended dose  Percentage of correct initial dosing interval  Incidence of nephrotoxicity | 80% vs. 40% (P < 0.001)  87% vs. 63% (P < 0.001)  17% vs. 25% (NS) | No | Yes | No |
| Day F (1995) | ED | 1 site; 465 patients | CBA | Guideline-driven electronic charting system that provided recommendations for evaluation and treatment of patients with acute low back pain | Mean charges | $257 vs. $239 (NS) | No | No | No |
| Dexter PR (2001) | Hospital | 1 site; 6371 patients | RCT | Computer generated reminders of patient eligibility for preventive care measures including prophylactic heparin use in high risk patients | Rate of prophylactic heparin use in high risk patients | 32.3% vs. 18.9% (P < 0.001) | No | Yes | No |
| Drescher FS (2011) | ED | 1 site; 434 patients | CBA | CDS system intended to improve the positive yield rate of CT angiography for evaluation of suspected PE | PE positive yield rate | 12.7% vs. 8.3% (NS) | No | No | No |
| Durieux P (2000) | Hospital | 1 site; 1971 patients | CBA | CDS system for provision of pharmacological VTE prophylaxis recommendations to orthopedic providers for post-operative patients | Rate of compliance with VTE prophylaxis guidelines | 94.9% (95% CI: 92.5%-96.6%) vs. 82.8% (95% CI: 77.6%-87.1%) | No | Yes | No |
| East TD (1999) | ICU | 10 sites;  200 patients | RCT | CDS system for management of mechanical ventilation | ICU length of stay | 27.6 vs. 25.2 days (NS) | No | No | No |
| Eden A (2009) | Hospital | 1 site; 995 patients | CBA | CDS system designed to remind anesthesiologists to reactivate alarms following cardiopulmonary bypass | Rate of alarm reactivation for 1^st^ intervention period  Rate of alarm reactivation for 2^nd^ intervention period | 63% vs. 22% (P < 0.001)  83% vs. 22% (P < 0.001) | No | Yes | No |
| Elkin PL (2010) | Hospital | 1 site; 1737 cases | CBA | Computer-based diagnostic decision support system made available to residents in a teaching hospital | Mean cost per diagnostically challenging case  Mean total charges per diagnostically challenging case  Mean Medicare Part A charges per diagnostically challenging case  Length of Stay | $7382 vs. $8318 (P = 0.0054)  $11403 vs. $12684 (P = 0.0180)  $9390 vs. $10422 (P = 0.0173)  3.99 vs. 4.14 days (NS) | Yes | Yes | Yes |
| Eslami S (2009) | ICU | 1 site; 696 patients | CBA | CDS intended to promote adherence to tidal volume recommendations in patients receiving mechanical ventilation | Average number of excessive tidal volume measurements per patient  Percentage of ventilation time with excessive tidal volume | 1.79 vs. 1.90 measurements (NS)  63.9% vs. 66.1% (NS) | No | No | No |
| Evans RS (1998) | ICU | 1 site;  1681 patients | CBA | Comprehensive antimicrobial management system in an ICU setting | Mean total cost of hospitalization when computer regimen was followed  Mean cost of antiinfective agents when computer regimen was followed  Total hospital length of stay when computer regimen was followed | $26315 vs. $35283 (P < 0.001)  $102 vs. $340 (P < 0.001)  10.0 vs. 12.9 days (P < 0.001) | Yes | Yes | No |
| Fernandez Perez ER (2007) | ICU | 1 site; 2200 patients | CBA | CPOE with CDS for management of RBC transfusions in the ICU setting | RBC transfusion cost  Average number of units transfused per patient | $556226 vs. $616442 (P = N/A)  1.3 vs. 1.5 units (P = 0.045) | Yes | Yes | No |
| Fischer MA (2003) | Hospital | 1 site; 1045 orders in intervention phase | CBA | CDS within CPOE system to identify and inform providers of patients who are eligible to transition from intravenous to oral route for five targeted medications | Change in IV defined daily dose  Change in PO defined daily dose  Change in length of stay  Change in total drug expenditures | -11.1% (P = 0.002)  +3.7% (P = 0.002)  +1.9% (P = N/A)  +12% (P = N/A) | Yes | No | No |
| Fitzgerald M (2011) | ED | 1 site;  1171 cases | RCT | CDS system to promote adherence to trauma protocols in the ED setting | Hospital length of stay  Average ICU length of stay  Error rate per patient  Percent of patients who received packed red blood cells | NS  70 vs. 112 hours (NS)  2.13 vs. 2.30 errors per patient (P = 0.04)  66.1% vs. 84.9% (P < 0.001) | No | Yes | No |
| Flanders SJ (2009) | ICU | 2 sites; 1482582 glucose measures | CBA | CDS system designed to prevent hyperglycemia in patients receiving IV insulin in the ICU setting | Odds of a glucose measure > 150 mg/dL during 1^st^ year post-baseline  Odds of a glucose measure > 150 mg/dL during 2^nd^ year post-baseline  Odds of a glucose measure >150 mg/dL during 3^rd^ year post-baseline | OR = 1.8, 95% CI = 1.78-1.82  OR = 2.16, 95% CI = 2.14-2.19  OR = 2.28, 95% CI = 2.25-2.30 | No | Yes | No |
| Fogel SL (2013) | ICU | 1 site; 1682 patient admissions | CBA | CDS system to guide intravenous insulin dosing in critically ill surgical patients | Mean percent decrease in the frequency of hyperglycemia measurements  Percent reduction in severe hypoglycemic measurements | 50% (P < 0.0001)  95% (P < 0.0001) | No | Yes | No |
| Galanter WL (2004) | Hospital | 1 site; 1596 alerting situations | CBA | CPOE with CDS designed to improve the safe use of digoxin by providing alerts related to digoxin, potassium, and magnesium serum levels | Unknown serum values checked within one hour  Unknown serum values checked within 24 hours  Low serum values supplemented within one hour  Low serum values supplemented within 24 hours | Improved with P < 0.01 for 3 of 3 measures  Improved with P < 0.01 for 3 of 3 measures  Improved with P < 0.05 for 3 of 4 measures  Improved with P < .05 for 2 of 4 measures | No | Yes | No |
| Galanter WL (2005) | Hospital | 1 site; 410 alerts or alerting situations | CBA | CPOE with CDS designed to alert providers attempting to order medication contraindicated for patients with renal insufficiency | Likelihood of patient receiving at least one dose of contraindicated medication | 47% vs. 89% (P < 0.0001) | No | Yes | No |
| Galanter WL (2010) | Hospital | 1 site; 38647 patients | CBA | Mandatory VTE risk assessment form integrated into the hospital’s CPOE system. System provided recommendations for pharmacological prophylaxis based on risk | Overall VTE rates  Rate of pharmacological prophylaxis | 0.43% vs 0.51% (NS)  36.8% vs. 25.9% (P < 0.0001) | No | Yes | No |
| Giuliano KK (2011) | ICU | 2 sites; 135 patients | CBA | CDS system designed to increase adherence to sepsis protocols in the ICU setting | Total hospital length of stay  ICU length of stay | 17.8 vs. 15.7 days (NS)  10.3 vs. 7.4 days (NS) | No | No | No |
| Griffey RT (2012) | ED | 1 site; 1407 patients | CBA | CPOE with CDS designed to guide medication dosing in geriatric patients in an ED setting | Proportion of orders consistent with dosing recommendations  Proportion of patients who experienced an ADE  ED length of stay | 31.4% vs. 23% (P < 0.001)  3.4% vs. 7.1% (P = 0.02)  5.6 vs. 5.8 hours (NS) | No | Yes | No |
| Haut ER (2012) | Hospital | 1 site; 1599 patients | CBA | CPOE with CDS designed to identify patients at high risk for VTE and recommend prophylaxis | Rate of preventable harm from VTE  Rate of compliance with guideline-appropriate prophylaxis | 0.17% vs. 1.0% (P = 0.04)  84.4% vs. 66.2% (P < 0.001) | No | Yes | No |
| Hoekstra M (2010) | ICU | 1 site; 2210 patients | CBA | CDS system to support the dosing potassium | Incidence of hypokalemia  Incidence of hyperkalemia  Average length of ICU stay  Average length of hospital stay | 1.7% vs. 2.4% (P < 0.001)  4.8% vs. 7.4% (P < 0.001)  NS  NS | No | Yes | No |
| Hulgan T (2004) | Hospital | 1 site; 15194 orders | CBA | CDS to promote the use of oral—versus IV—quinolones | Percent of quinolone orders for oral route | 62.4% vs. 55.5% (P <= 0.001 ) | No | Yes | No |
| Jones S (2011) | Hospital | 1 site; 1481 patients | CBA | CDS to alert physicians to patients at early risk for deterioration | Average hospital length of stay | 6.9 vs. 9.7 days (P < 0.001) | No | Yes | No |
| Kazemi A (2011) | Hospital | 1 site; 248 patients | CBA | CPOE with CDS designed to address medication dosing errors in a neonatal ward setting | Medication error rate | 34% vs. 53% (P < 0.001) | No | Yes | No |
| Kellet J (2001) | ICU | 1 site; 894 patients | CBA | CDS system designed to aid in the decision to treat cases of acute myocardial infarction with fibrinolytic therapy | Proportion of appropriate candidates who received fibrinolytic therapy | 68.9% vs. 66.7% (NS) | No | No | No |
| Knirsch CA (1998) | Hospital | 1 site; 43 patients | NRCT | Automated computer protocol for identification of patients requiring tuberculosis isolation combined with a clinical protocol | Isolation of culture positive tuberculosis patients within 24 hours of admission | 79% vs. 70% (NS) | No | No | No |
| Kucher N (2005) | Hospital | 1 site; 2506 patients | RCT | CDS system to identify patients at risk for deep venous thrombosis and alert physicians | Rate of venous thromboembolism at 90 days  Rate of pulmonary embolism at 90 days  Proportion of patients who received mechanical prophylaxis  Proportion of patients who received pharmacological prophylaxis | 4.9% vs. 8.2% (P = 0.001)  1.1% vs. 2.8% (P = 0.004)  10% vs. 1.5% (P < 0.001)  23.6% vs. 13.0% (P < 0.001) | No | Yes | No |
| Larsen RA (1989) | Hospital | 1 site; 6831 patients | CBA | Computer generated paper reminders of need for perioperative antibiotics | Surgical wound infection rates  Percent of eligible patients who received perioperative antibiotics | 0.9% vs. 1.8% (P < 0.03)  58% vs. 40% (P < 0.001) | No | Yes | No |
| Lecumberri R (2008) | Hospital | 1 site; 19338 patients | CBA | CDS system for identification of patients at high risk for VTE and notification of physicians of the need for prophylaxis | VTE rate for 1^st^ intervention period  VTE rate for 2^nd^ intervention period | 1.74 vs. 3.26 events/1000 patients (P < 0.05)  1.67 vs. 3.26 events/1000 patients (P < 0.05) | No | Yes | No |
| Levick DL (2013) | Hospital | 3 sites; 41306 admissions | ITS | CPOE with CDS intended to prevent the ordering of unnecessary BNP tests | Number of patients receiving two or more BNP tests during the same admission  Decrease in supply costs per year | 487 vs. 1358 patients (P < 0.01)  $92,000 (P = NR) | No | Yes | No |
| Lipton JA (2011) | ICU | 1 site; 667 patients | CBA | CDS system intended to assist with the management of intravenous insulin therapy in a coronary critical care unit | Proportion of patients with a mean blood glucose level within the target range  Length of stay per patient | 43% vs. 31% (P = 0.01)  1.00 vs. 1.05 days (NS) | No | Yes | No |
| Maat B (2013) | ICU | 1 site; 2040 patients | ITS | CDS system designed to assist calculation of glucose in parenteral nutrition for neonates | Mean incidences of hyperglycemias per 100 hospital days in every 3 month period  Mean incidences of hypoglycemias per 100 hospital days in every 3 month period | 5.0 vs. 6.0 incidences per 100 hospital days (NS)  3.1 vs. 4.0 incidences per 100 hospital days (NS) | No | No | No |
| Mann EA (2011) | ICU | 1 site; 18 patients | RCT | CDS intended to support the management of insulin in burn patients in the ICU setting | Percent of time in target glucose range | 47% vs. 41% (p < 0.05) | No | Yes | No |
| Matsumura Y (2009) | Hospital | 1 site; 970 patients | CBA | CDS implemented with CPOE to prevent the ordering of contraindicated medications for patients with renal insufficiency | Rate of discontinuation of contraindicated medication | 54% vs. 24% (P = 0.01) | No | Yes | No |
| McCoy AB (2010) | Hospital | 1 site; 1659 patients | CBA | CDS system integrated in CPOE system to improve medication ordering for patients with acute kidney injury | Rate of modification or discontinuation of contraindicated drugs | 52.6 vs. 35.2 /100 events (P < 0.001) | No | Yes | No |
| McGregor JC (2006) | Hospital | 1 site | RCT | CDS to provide antimicrobial management support to the institution’s antimicrobial management team | Average antimicrobial expenditures per patient  Length of stay | $127.77 vs. $163.00 (P < 0.05)*  3.84 vs. 3.99 days (NS) | Yes | Yes | No |
| McKinley BA (2001) | ICU | 1 site; 67 patients | RCT | CDS system to support management of mechanical ventilation in trauma-induced acute respiratory distress syndrome | ICU length of stay  Barotrauma score | 34.5 vs. 31.4 days (NS)  1.01 vs. 0.83 (NS) | No | No | No |
| Mekhjian HS (2002) | Hospital | 2 sites; 28898 patients | CBA | Comprehensive CPOE system with CDS for pharmacotherapy | Severity-adjusted total cost per admission at hospital #1  Severity-adjusted total cost per admission at hospital #2  Severity-adjusted length of stay at hospital #1  Severity-adjusted length of stay at hospital #2 | $5661 vs. $5697 (NS)  $6518 vs. $6427 (NS)  3.71 vs. 3.91 days (P < 0.002)  3.61 vs. 3.68 (NS) | Yes | No | No |
| Meyfroidt G (2011) | ICU | 1 site; 1373 patients | CBA | CDS system to support the management of IV insulin in an ICU setting | Proportion of blood glucose measurements in the hyperglycemic range  Proportion of patients that experienced at least one episode of hypoglycemia | 30% vs. 32.9% (P = 0.008)  4.0% vs. 6.5% (P = 0.043) | No | Yes | No |
| Milani RV (2011) | Hospital | 1 site; 80 patients | NRCT | CPOE with CDS to assist the prescription of antithrombotics in patients with chronic kidney disease admitted for acute coronary syndrome | Proportion of patients who received contraindicated antithrombotics  Proportion of patients who experienced in-hospital bleeding  Length of stay | 0% vs 17% (P = 0.01)  9% vs. 21% (NS)  4.8 vs. 9.1 days (P = 0.01) | No | Yes | No |
| Milani RV (2012) | Hospital | 1 site; 1321 patients | NRCT | CDS integrated into CPOE to support admission orders for patients with acute coronary syndrome | Length of stay  Percent of patients who received perfect care (met all quality measures for acute coronary syndrome care) | 3.5 vs. 3.8 days (P = 0.262)  89% vs. 61% (P < 0.001) | No | Yes | No |
| Mullett CJ (2001) | ICU | 1 site; 1758 patients | CBA | Comprehensive antimicrobial management system in a pediatric ICU setting | Mean hospital costs per patient  Total antiinfectives cost  Total PICU antiinfectives cost  PICU length of stay  Hospital length of stay | $28257 vs. $25032 (NS)  $289.60 vs. $274.79 (NS)  $183.53 vs. $177.03 (NS)  4.90 days vs. 4.93 days (NS)  10.76 vs. 10.76 days (NS) | Yes | No | No |
| Nash IS (2005) | Hospital | 1 site | CBA | CDS to identify patients with renal impairment who had received excessive dosage of medication. Physicians were then notified by nursing staff in one intervention period and notified by pharmacists in the other intervention period | Rate of excessive dosing with nurse feedback  Rate of excessive dosing with pharmacist feedback | 17.3% vs. 23.2% (P < 0.05)  16.8% vs. 23.2% (P < 0.05) | No | Yes | No |
| Niemi K (2009) | Hospital | 1 site; 4090 patients | CBA | CDS system for identification of patients with pneumonia or heart failure and recommendations to providers to meet quality indicators | Compliance with quality indicators for pneumonia and heart failure cases | One of six measures improved with statistical significance | No | No | No |
| Paul M (2006) | Hospital | 3 sites; 2326 patients | RCT | CDS system designed to support the appropriate prescription of empiric antibiotics | Rate of appropriate empirical antibiotic treatment  Average total antibiotic costs  Mean length of stay | 72.7% vs. 64.5% (P = 0.033)  565.4 vs. 623.2 Euros (P = 0.007)  8.83 vs. 9.45 days (NS) | Yes | Yes | No |
| Peterson JF (2005) | Hospital | 1 site; 3718 patients | CBA | CPOE with CDS to guide the prescription of psychotropic medications for geriatric patients | Median length of stay  Fall injuries per 100 patient-days | 4 vs. 4 days (NS)  0.06 vs. 0.17 (NS) | No | No | No |
| Potts AL (2004) | ICU | 1 site; 514 patients | CBA | CPOE with CDS in a pediatric ICU setting | Potential ADE rate  Medication prescribing error rate  Rule violation rate | 1.3 vs. 2.2 events per 100 orders (P <0.001)  0.2 vs. 30.1 events per 100 orders (P < 0.001)  0.1 vs. 6.8 events per 100 orders (P < 0.001) | No | Yes | No |
| Qian Q (2011) | Hospital | 1 site | CBA | CDS system intended to alert physicians to patients with left ventricular systolic dysfunction who were not being treated with an ACEi/ARB agent | Percentage of ACEi/ARB adherence | 97.6% vs. 88.4% (P < 0.01) | No | Yes | No |
| Raja AS (2012) | ED | 1 site; 6838 patients | CBA | CDS system intended to assist with the use of CT pulmonary angiography for acute pulmonary embolism | Quarterly utilization of CT pulmonary angiography | 21.1 vs. 26.4 examinations/1000 patients (P = 0.0379) | No | Yes | No |
| Riggio JM (2009) | Hospital | 1 site; 65604 admissions | CBA | CDS system to assist in the early detection of HIT | Average time from fall in platelet count to heparin-induced thrombocytopenia testing  Average time from fall in platelet count to heparin-induced thrombocytopenia therapy  Average time from fall in platelet count to discontinuation of heparin products | 3.0 vs. 2.3 days (NS)  15.0 vs. 19.3 days (NS)  2.9 vs. 1.3 days (p = 0.04) | No | No | No |
| Rind DM (1991) | Hospital | 1 site; 10076 patients | CBA | CDS designed to alert physicians to rising creatinine levels in patients taking nephrotoxic drugs | Mean time from rise in creatinine to discontinuation or dose adjustment of nephrotoxic drug | 72.6 vs. 93.7 hours (P < 0.001) | No | Yes | No |
| Roberts GW (2010) | Hospital | 1 site; 1001 patients | CBA | CDS system for recommendations on appropriate prescription of certain renally cleared drugs in patients with renal insufficiency | Enoxaparin dosing conformant with guideline  Gentamicin dosing conformant with guideline  Vancomycin dosing conformant with guideline  Rate of holding renally cleared drugs during periods of acute renal failure | 86% vs. 68% (P = 0.03)  87% vs. 63% (P = 0.01)  77% vs. 47% (NS)  62% vs. 38% (p = 0.01) | No | Yes | No |
| Rood E (2005) | ICU | 1 site; 120 patients | RCT | CDS system designed to support insulin dosing in an ICU setting | Proportion of time spent in target glucose range | 54.2% vs. 52.9% (difference of 1.3, 95% CI = 1.0-1.56) | No | No | No |
| Rothschild JM (2007) | Hospital | 1 site; 1607 patients | RCT | CDS for management of blood product transfusions | Percent of transfusion orders which were not guideline compliant (typically unnecessary or excessive) | 59.6% vs. 67.5% (P < 0.0001) | No | Yes | No |
| Roukema J (2008) | ED | 1 site; 164 patients | RCT | CDS system for the evaluation/management of children with fever without apparent cause in the ED setting | Median length of ED stay  Frequency of lab orders | 138 vs. 123 minutes (NS)  82% vs. 44% (P < 0.001) | No | No | No |
| Salinas J (2011) | ICU | 1 site; 105 patients | CBA | CDS system designed to support the fluid resuscitation of severely burned patients in the ICU setting | Mean ventilator free days  Mean ICU free days | 6.5 vs. 3.8 days (P < 0.05)  NS | No | Yes | No |
| Schriger DL (1997) | ED | 1 site; 206 patients | CBA | Guideline-driven electronic charting system that provided recommendations for evaluation and treatment of occupational exposure to blood or body fluids | Charges per patient | $384 (intervention)vs. $460 (baseline) (95% CI $54 to $184 difference in charges) | No | Yes | No |
| Schriger DL (2000) | ED | 1 site; 830 patients | CBA | Guideline-driven electronic charting system that provided recommendations for evaluation and treatment of febrile children less than 3 years old | Median charges  Mean charges | $216 (intervention) vs. $216 (baseline) vs. $222 (post-intervention) (NS)  $387 (intervention) vs. $357 (baseline) vs. $635 (post-intervention) (NS) | No | No | No |
| Sellier E (2009) | Hospital | 1 site; 603 patients | CBA | CPOE with CDS intended to decrease inappropriate prescriptions in patients with renal insufficiency | Proportion of prescriptions which were inappropriate | 19.9% vs. 21.3% (NS) | No | No | No |
| Tafelski S (2010) | ICU | 1 site; 186 patients | CBA | CDS intended to assist in the management of sepsis and septic shock in an ICU setting | Concordance with sepsis management guidelines  Mean daily antibiotic usage  Percent of ICU days which were antibiotic-free | 90.8% vs. 39.8% (P < 0.05)  1.3 vs 1.5 agents/day (P < 0.05)  25% vs. 18.4% (P < 0.05) | No | Yes | No |
| Teich JM (2000) | Hospital | 1 site; 6452 orders | CBA | CPOE with embedded CDS to promote the use of heparin in patients with an order for bed rest | Proportion of bed rest orders accompanied by a heparin order | 46.9% vs. 23.9% (P < 0.001) | No | Yes | No |
| Thursky KA (2006) | ICU | 1 site; 1060 admissions | CBA | CDS for antibiotic management in an ICU setting | Odds patient prescribed carbapenems after adjusting for risk factors  Odds patient prescribed 3^rd^ generation cephalosporin after adjusting for risk factors  Odds patient prescribed vancomycin after adjusting for risk factors | OR = 0.61, 95% CI = 0.39-0.97  OR = 0.58, 95% CI = 0.42-0.79  OR = 0.67, 95% CI = 0.45-1.00 | No | Yes | No |
| Vardi A (2007) | ICU | 1 site;  60094 orders | CBA | CDS designed to prevent medication errors for resuscitation drugs used in a pediatric critical care setting | Number of medication order errors | 0 vs. 3 medication order errors (P = N/A) | No | No | No |
| Wang H (2012) | Hospital | 1 site; 38647 prescriptions | CBA | CDS within CPOE system intended to prevent dosing errors of 13 antibiotics which require adjustment based on creatinine clearance | Percent of antibiotic prescriptions with an inappropriate dosage during the 4^th^ year following implementation  Incidence rate of renal function deterioration during the 4^th^ year following implementation | 3.42% vs. 21.3% (P < 0.001)  9.47% vs. 10.94 (P < 0.001) | No | Yes | No |

ACEi = angiotensin-converting-enzyme inhibitor; ADE = adverse drug event; AKI = acute kidney injury; ARB = angiotensin receptor blocker; BNP = B-type natriuretic peptide; CBA = controlled before-after; CDS = clinical decision support; CPOE = computerized provider order entry; CT = computed tomography; ED = emergency department; HIT = heparin-induced thrombocytopenia; ICU = intensive care unit; ITS = interrupted-time-series; IV = intravenous; NR = not reported; NRCT = non-randomized controlled trial; NS = not significant; OR = odds ratio; PE = pulmonary embolism; RBC = red blood cell; RCT = randomized controlled trial; VTE = venous thromboembolism

^*^P value not provided by manuscript; calculated using raw data provided in manuscript

^#^P value not provided by manuscript; calculated using raw data provided in manuscript; assumes historical control period had same sample size as intervention period
